# Systematic endoscopic staging of mediastinum to determine impact on radiotherapy for locally advanced lung cancer (SEISMIC): protocol for a prospective single arm multicentre interventional study

**DOI:** 10.1186/s12890-022-02159-9

**Published:** 2022-09-24

**Authors:** Daniel P. Steinfort, Shankar Siva, Kanishka Rangamuwa, Percy Lee, David Fielding, Phan Nguyen, Barton R. Jennings, Shaun Yo, Nick Hardcastle, Gargi Kothari, Laurence Crombag, Jouke Annema, Kazuhiro Yasufuku, David E. Ost, Louis B. Irving

**Affiliations:** 1 Department of Respiratory and Sleep Medicine, Royal Melbourne Hospital, 300 Grattan Street, VIC, Melbourne, 3050 Australia; 2 Department of Oncology, Peter MacCallum Cancer Centre, Melbourne, Australia; 3 Department of Radiation Oncology, MD Anderson Cancer Center, The University of Texas, Houston, USA; 4 Department of Thoracic Medicine, Royal Brisbane and Women’s Hospital, Brisbane, Australia; 5 Department of Thoracic Medicine, Royal Adelaide Hospital, Adelaide, Australia; 6 Department of Lung and Sleep, Monash Health, Melbourne, Australia; 7 Department of Pulmonology (VUmc), University of Amsterdam, Amsterdam, The Netherlands; 8 Department of Pulmonology (AMC), University of Amsterdam, Amsterdam, The Netherlands; 9 Division of Thoracic Surgery, Toronto General Hospital, Toronto, Canada; 10 Department of Pulmonary Medicine, MD Anderson Cancer Center, The University of Texas, Houston, USA

**Keywords:** Bronchoscopy, Cancer staging, Carcinoma, Non-small-cell lung, Endoscopic ultrasonography

## Abstract

**Background:**

Endobronchial ultrasound-guided transbronchial needle aspiration (EBUS-TBNA) is established as the preferred method of mediastinal lymph node (LN) staging in non-small cell lung cancer (NSCLC). Selective (targeted) LN sampling is most commonly performed however studies in early stage NSCLC and locally advanced NSCLC confirm systematic EBUS-TBNA evaluation improves accuracy of mediastinal staging. This study aims to establish the rate of detection of positron emission tomography (PET)-occult LN metastases following systematic LN staging by EBUS-TBNA, and to determine the utility of systematic mediastinal staging for accurate delineation of radiation treatment fields in patients with locally advanced NSCLC.

**Methods:**

Consecutive patients undergoing EBUS-TBNA for diagnosis/staging of locally advanced NSCLC will be enrolled in this international multi-centre single arm study. Systematic mediastinal LN evaluation will be performed, with all LN exceeding 6 mm to be sampled by TBNA. Where feasible, endoscopic ultrasound staging (EUS-B) may also be performed. Results of minimally invasive staging will be compared to FDG-PET. The primary end-point is proportion of patients in whom systematic LN staging identified PET-occult NSCLC metastases. Secondary outcome measures include (i) rate of nodal upstaging, (ii) false positive rate of PET for mediastinal LN assessment, (iii) analysis of clinicoradiologic risk factors for presence of PET-occult LN metastases, (iv) impact of systematic LN staging in patients with discrepant findings on PET and EBUS-TBNA on target coverage and dose to organs at risk (OAR) in patients undergoing radiotherapy.

**Discussion:**

With specificity of PET of 90%, guidelines recommend tissue confirmation of positive mediastinal LN to ensure potentially early stage patients are not erroneously denied potentially curative resection. However, while confirmation of pathologic LN is routinely sought, the exact extent of mediastinal LN involvement in NSCLC in patient with Stage III NSCLC is rarely established. Studies examining systematic LN staging in early stage NSCLC report a significant discordance between PET and EBUS-TBNA. In patients with locally advanced disease this has significant implications for radiation field planning, with risk of geographic miss in the event of PET-occult mediastinal LN metastases. The SEISMIC study will examine both diagnostic outcomes following systematic LN staging with EBUS-TBNA, and impact on radiation treatment planning.

***Trial registration*:**

ACTRN12617000333314, ANZCTR, Registered on 3 March 2017.

## Background

Endobronchial ultrasound-guided transbronchial needle aspiration (EBUS-TBNA) is a widely accepted technique for minimally invasive sampling of mediastinal lymph nodes (LN). It is the preferred method for staging of patients presenting with suspected locally advanced lung cancer due to its excellent diagnostic accuracy and safety profile [1]. Non-invasive imaging with positron emission tomography (PET), generally performed pre-procedurally, is used to complete clinical staging. Stage III NSCLC is suspected following demonstration of fluoro-deoxyglucose (FDG) avidity in mediastinal LN.

Accuracy of PET for detection of metastatic LN in NSCLC patients is imperfect [2] and consequently international clinical practice guidelines mandate biopsy confirmation of PET-positive mediastinal LN [1, 3, 4]. In this scenario, “selective” sampling with EBUS-TBNA is generally targeted to pathologic (FDG-avid) stations with the primary intent of confirming a pathologic diagnosis. If metastatic involvement is confirmed at any site no further sites are examined. Confirmation of metastatic involvement commonly results in a recommendation of definitive chemoradiotherapy. Despite the known imperfect accuracy of PET for evaluation of mediastinal LN in NSCLC, radiation target volumes are most commonly based on PET-identified extent of disease [5].

Systematic mediastinal LN staging in early stage NSCLC prior to surgical resection is recommended in patients with risk factors for post-operative up-staging [3]. Systematic EBUS-TBNA staging in early stage NSCLC may identify PET-occult mediastinal LN metastases in up to 1 in 14 patients. Our single centre pilot study confirmed that discordant findings between PET-CT and EBUS-TBNA regarding extent of mediastinal LN involvement are seen in a significant proportion of patients with clinical stage III NSCLC, with both up-staging (i.e. detection of PET-occult LN metastases) and down-staging observed [6]. Follow-on studies also confirmed that EBUS-TBNA prior to curative-intent radiotherapy significantly improves coverage of subclinical disease through detection of PET-occult metastases. Identification of false-positive lymph node involvement in highly selected cases may reduce radiation dose to organs at risk (OAR) [7].

This prospective international multi-centre study will examine the use of systematic mediastinal LN staging in patients with locally advanced (stage III) NSCLC. Aims of the study include:

1. To determine the rate of detection of PET-occult LN metastases in patients with locally advanced (Stage III) NSCLC
2. To identify the proportion of patients with potentially false positive involvement of mediastinal LN on PET
3. To identify clinical risk predictors for presence of PET-occult LN metastases
4. In patients with discrepant findings on PET and EBUS-TBNA, to compare target coverage and radiation dose to OARs using target volumes based on PET alone *versus* PET and EBUS-TBNA.

### Importance of comprehensive mediastinal lymph node staging in early stage NSCLC

The importance of accurate LN staging in early stage NSCLC is well established. Guidelines recommend systematic LN staging at the time of curative intent surgery [4, 8, 9], both to ensure complete resection (R0) of all disease, and to ensure accurate disease staging for optimal guidance of adjuvant therapy recommendations. The importance of accurate LN staging may be illustrated by poorer survival outcomes in patients undergoing surgery where resection is considered incomplete, due either to lack of systematic nodal dissection, or positivity of the highest mediastinal node removed [10, 11]. Critically, 5-year survival in patients where the highest LN removed was positive was markedly worse than in patients with presence of carcinoma in situ at the bronchial resection margin (29 vs 40%) [10]. The International Association for the Study of Lung Cancer identify the significance of have described the significance of LN staging for defining the residual tumour descriptor in resected NSCLC [12], and assigned patients with metastatic involvement of the highest mediastinal LN resected as having an unknown residual tumour status (R(un)), with this group experiencing significantly worse median survival than in patients in whom adequate nodal staging is completed.

Variability in adequacy of LN examination/sampling for resected NSCLC has been suggested to explain a large proportion of intercontinental survival differences [11]. Consequently, the National Comprehensive Cancer Network recommends a minimum of 3 or more mediastinal nodal stations, the American College of Surgeons Commission on Cancer recommends 10 total LN regardless of station, and the Union for International Cancer Control seventh edition recommends 6 total nodes, 3 from N1 and 3 from N2 stations are sampled [9].

Accuracy of EBUS-TBNA for systematic LN staging in early stage NSCLC is established, though is recognized to be inferior to that observed for patients with radiologically pathologic mediastinal LN [13] EBUS-TBNA may demonstrate PET-occult disease in up to 10% of NSCLC patients with a radiologically normal mediastinum, and up to 29% of patients staged cN1 on PET/CT [14–16]. International guidelines recommend EBUS-TBNA staging pre-operatively in patients with radiologic features associated with an increased risk of post-operative pathologic upstaging [3]

### Comprehensive mediastinal lymph node staging in locally advanced NSCLC

In contrast to early stage NSCLC where intra-operative LN staging is recommended to achieve completion of pathologic staging, current International guidelines recommend radiation target volumes be constructed based on PET-identified extent of disease [5, 17] In this setting, any PET-occult LN metastases would not be included in the radiation field [18] and potentially result in a higher likelihood of regional failure. Equally, inclusion of false positive LN may result in unnecessary increased radiation dose to critical structures, with consequent increased risk of organ toxicity [7]

Incomplete staging of the mediastinum in patients with locally advanced NSCLC is analogous to R(un) staging described above in patients undergoing resection—while many patients will have the entirety of their disease encompassed by radiation fields, an unknown proportion (estimated 5–29% based on previously published studies [6, 15, 19] will have disease located outside the radiation field. Patients whose disease by definition has demonstrated metastatic potential to LN may even be expected to have a higher rate of presence of PET-occult disease—a higher rate of postoperative upstaging to pN2 is observed in patients with cN1 disease, and a similar higher rate of pN2 disease has been observed following systematic EBUS-TBNA mediastinal staging in NSCLC [15]

Similar to studies in early stage NSCLC, our single centre pilot study [6], consistent with prior retrospective studies [19, 20] confirmed a significant rate of detection of PET-occult LN metastases in NSCLC following systematic mediastinal staging with EBUS-TBNA. Critically, our follow-up study confirmed that this has major implications for radiotherapy planning and probability of tumour control following curative-intent radiation [7] predominantly due to reduction of geographic miss in these patients.

EBUS is now considered standard of care in management of lung cancer. The utility of Endoscopic Ultrasound (EUS) in mediastinal staging of lung cancer has long been established, with complementary reach of the techniques allowing a greater number of LN stations to be assessed via minimally invasive means. More recently use of the endobronchial video-bronchoscope has allowed “complete” mediastinal staging. EUS using the bronchoscope (EUS-B) can be performed by pulmonologists in the same procedure as EBUS-TBNA [21, 22] and multiple studies have confirmed that **t**he combined use of EBUS-TBNA and EUS-FNA significantly improves sensitivity in detecting mediastinal nodal metastases in NSCLC [13, 23] Consequently, where possible, European Respiratory Society and European Society of Thoracic Surgeons guidelines on staging of lung cancer recommend both techniques be employed [3]

## Methods/design

*Study design *The SEISMIC study (**S**ystematic **E**ndoscop**I**c **S**taging of **M**ediastinum to determine **I**mpact on radiotherapy for locally advanced lung **C**ancer) is a prospective international multi-centre single arm interventional study.

*Setting* seven international tertiary lung cancer services.

*Intervention* systematic LN staging with EBUS-TBNA will be performed at the time of staging EBUS as previously described [6] At centres where expertise and experience allows, endoscopic staging will comprise both EBUS and EUS-B [22] mediastinal examination and sampling.

*Patient population* patients with known or suspected NSCLC and suspected or known mediastinal metastases (i.e. cN2/3 disease), based upon CT and PET findings. Inclusion and Exclusion criteria are recorded in Box 1.

**Table:** 

Box 1: Inclusion and exclusion criteria	
Inclusion criteria • Patients with known/suspected NSCLC • Patients with suspected or known mediastinal metastases (i.e. cN2/3 disease) • Patients are potential candidates for radical dose or high-dose palliative conventional radiotherapy (± chemotherapy)	Exclusion criteria • Medical co-morbidities preclude bronchoscopy • Medical co-morbidities or known pathologic extent of disease precludes consideration of radiotherapy • Age < 18 years

*Outcomes* the primary study outcome is proportion of patients in who PET-occult mediastinal LN metastases are detected by systematic LN staging with EBUS-TBNA/(EUS-B-FNA).

Secondary outcomes include:

- Proportion of patients with endoscopy-demonstrated benign LN at sites of FDG-avidity on PET/CT
- Multi-regression analysis of clinic- radiologic factors to identify risk factors for endoscopic detection of PET-occult disease
- Difference in planned radiation dose to lymph nodes and adjacent critical organs in treatment plans based on FDG-PET/CT only vs FDG-PET/CT & EBUS-TBNA in patients where EBUS-TBNA staging demonstrates discrepant extent of mediastinal involvement compared with FDG-PET/CT.
- Radiomics analysis to identify risk factors for EBUS-PET discrepancy regarding extent of mediastinal LN involvement.
- Incidence of detection of PET-occult disease by EUS-B-FNA
- Development of a radiomics signature to predict nodal status as defined by EBUS
- Outcomes of surgical staging (where performed) in patients with PET + /EBUS– findings

### Data collection

All patients’ demographic and clinical data will be collected including gender, age, smoking status, whether symptomatic disease, and histology.

Radiologic features from PET-CT scans include primary tumour size, peripheral *vs* central position (“concentric” lines, inner one-third, centre of the tumour, as per Casal et al. [24]) of primary tumour, N-stage as determined by CT chest, N-stage as determined by FDG-PET, and highest LN station involved on radiologic imaging.

EBUS imaging features; including whether endobronchial tumour was seen [25] and which PET negative nodes were identified, which nodes were sampled, station, sonographic features of LN (size, LN margin, shape, echogenicity, central hilum visible, necrosis sign visible), number of LN sampled, whether EUS-B was performed and EUS-B features, procedure time and procedural complications.

Additionally, collection of data will include; final N-stage as determined by EBUS-TBNA/EUS-B, highest LN station involved by NSCLC, and any discrepancy between PET and EBUS-TBNA staging.

### Performance of systematic LN staging

#### PET-CT

Integrated PET-CT scans will be performed before recruitment into the study and performance of EBUS staging bronchoscopy. Pre-treatment staging established according to the 8th edition of the Lung Cancer Stage Classification, the TNM descriptors for which are reviewed in detail elsewhere [26]

#### Endoscopic staging procedure

Endoscopic staging will be performed according to the following methodology:

- Visual examination of the following LN stations is required in all cases – 2R, 2L, 4R, 4L, 7. In centres where EUS-B is utilized, endoscopic examination also requires visualization of stations 4L, 5, 7, 8, 9.
- Any visible LN exceeding 6 mm in these stations will be sampled via EBUS-TBNA and/or EUS-B-FNA. Rapid on-site cytology examination (ROSE) of TBNA/FNA aspirates will be used where available to confirm adequate specimens/diagnostic tissue is obtained before proceeding to the next LN station.
- Systematic sampling should commence at the highest echelon mediastinal LN visualized (ie. N3) and proceed more proximally, guided by ROSE results. Where ROSE was unavailable, PET-positive LN should be sampled a minimum of three times by TBNA. Assessment of contralateral hilar LN is not planned due to the impact on procedure time & complexity, given the exceedingly low expected rate of disease at this anatomic site [27]
- Samples per nodal station:
  - For PET-negative LN (low pre-test probability) where ROSE demonstrates adequate samples, no further sampling from that site will occur. We would plan to perform as few as 1 TBNA per LN station
  - For PET-positive LN (where pre-test probability of malignant involvement is higher), Three TBNA needle passes will be performed when ROSE indicates benign tissue, to ensure highest NPV at these high risk sites.
- At centres where expertise & experience allows, endoscopic staging will comprise both EBUS and EUS-B mediastinal examination & sampling. Remaining centres will undertake systematic sampling of mediastinal LN via EBUS-TBNA.

#### Dosimetry endpoints

This component is an in silico study that will examine dosimetric consequences of PET-EBUS-TBNA discrepancies observed. Clinical treatment plans will be constructed after active involvement in the study has been completed. Dosimetry of radiation treatment plans are routinely performed in all patients using standardised computer-based dosimetric calculations, which include routine dose/volume constraints such as the volume of lung that receives 5 Gray (Gy) (V5Gy), the V20Gy, V30Gy and mean lung dose. Clinical treatment plans are routinely constructed on the basis of PET-identified disease extent. Where Endosonographic findings suggest a different extent of mediastinal involvement, the dosimetric differences in lymph node and critical organ dose between treatment plans based on FDG-PET/CT only vs FDG-PET/CT & EBUS-TBNA staging will be calculated.

For study endpoints, two different scenarios will occur where dosimetric consequences of systematic endosonographic staging will be calculated. These will be theoretical in nature, comparing the radiation plan based upon PET based target volumes to a radiation plan based upon PET- and EBUS- based target volumes.

1. EBUS-TBNA identifies PET-occult disease (PET-/EBUS +): The clinical treatment plan will be constructed to include in the treatment field both the PET + and EBUS + mediastinal LN sites. The hypothetical plan will be based solely on PET + sites. The difference in radiation dose at PET-/EBUS + sites between the clinical and hypothetical plans will be determined. From this difference the risk of recurrence will be estimated according to published studies relating radiation dose to tumour control probability.
2. EBUS-TBNA demonstrates benign lymph node tissue in PET-positive lymph nodes (PET + /EBUS-): The clinical treatment plan will be constructed to include all PET + sites (due to the imperfect negative predictive value of endosonographic staging—approx 95%). The hypothetical plan will be based on the endosonographic-determined disease extent. The difference in radiation dose to critical organs including the lung, heart, oesophagus between the clinical and hypothetical treatment plans will be calculated. Estimates of toxicity probability will be computed based on existing relationships between organ dose and toxicity risk.

### Sample size calculation

Study sample size has been calculated at 190. This is based on the view of investigators regarding the expected primary outcome (proportion of participants with PET-occult disease detected by EBUS-TBNA) of 9–11 percent, based on previously published studies [6]

95% CI for this outcome with n = 190 is 7.3–16.4%.

### Data analysis

Primary study outcome will be reported using observed prevalence (%) with 95% confidence intervals.

Identification of clinicoradiologic risk factors for presence of PET-occult disease will be completed using multinomial or ordinal regression.

Application of established risk prediction tools for prediction of nodal disease in patients with NSCLC as determined by EBUS-TBNA [28] will also be performed for identification of patients with PET-EBUS discrepant findings.

Radiation dosimetry endpoints will be based on in silico radiation plans developed according to staging based on PET findings alone, versus PET + EBUS staging findings.

#### Radiomics analysis

Individual lymph node stations will be manually contoured on the diagnostic contrast enhanced CT and PET scans of all patients by a radiation oncologist and used as the volume of interest (VOI). Radiomic features will be extracted from the VOI using a specialized, validated, open source software (PyRadiomics) available on the python platform. Radiomics features extracted will include first order features, shape features and second order texture features, including grey scale co-occurrence matrix, neighbourhood grey tone difference matrix, grey level run-length matrix, grey level size zone matrix, and level dependence matrix. The association between lymph node status and radiomics features will be modelled using a variety of binary classifiers (such as logistic regression, random forests and support vector machines). Each of the classifiers will be employed within a nested ten-fold cross-validation framework and classifier accuracy will be reported with sensitivity, specificity, and area under the receiver operating characteristic curve (AUC).

## Discussion

Non-invasive staging is a critical step in assessment of patients with NSCLC. However the imperfect sensitivity of PET in mediastinal staging of NSCLC means (minimally) invasive confirmation of findings is frequently sought. With specificity of PET of 90%, guidelines recommend tissue confirmation of positive mediastinal LN to ensure potentially early stage patients are not erroneously denied potentially curative resection. However, while confirmation of pathologic LN is routinely sought, the extent of mediastinal evaluation during staging EBUS-TBNA varies considerably [29, 30], and the exact extent of mediastinal LN involvement in NSCLC in patient with Stage III NSCLC is rarely established. Consequently, a proportion of patients receiving radical radiotherapy may be at risk of geographic miss (in the event of PET-occult LN metastases) and higher rates of disease recurrence, or receive radiation to a larger field than is needed (in the event of false-positive PET findings) with consequent elevated toxicity risk in OAR. We believe this variance in care presents an important opportunity to improve clinical outcomes in NSCLC.

Systematic mediastinal LN staging with EBUS-TBNA in patients with early stage NSCLC has demonstrated a significant rate of detection of PET-occult disease [15] as well as down-staging [31, 32] Thus it is well established that a significant rate of discrepancy in mediastinal assessment of patients with NSCLC between PET/CT and EBUS-TBNA exists. Less established is the rate of discrepancy in patients with locally advanced/cN2 NSCLC.

We hypothesize that systematic mediastinal LN staging in patients with Stage III NSCLC will provide more accurate information on the extent of mediastinal LN involvement. The SEISMIC study will examine both the outcomes following systematic LN staging, as well as the impact on radiation treatment planning.

On the other hand, systematic mediastinal assessment with EBUS-TBNA as compared to selective sampling is a non-trivial extension of current practice. Additional procedural time and potential for complications must be outweighed by the clinical utility of the approach. It may be that the low-intermediate dose wash of current radiotherapy treatments may cover subclinical radiological disease sufficiently to achieve cancer control. The purpose of this study is to evaluate these possibilities in a rigorously conducted prospective trial.

Patterns of lymph node metastases, based on primary tumour position have been reported previously in surgical cohorts [33] as have patterns of locoregional relapse following definitive chemoradiation [34]. Depending on findings from this study, a more focused approach to LN sampling by EBUS-TBNA prior to definitive chemoradiation may be identified, based either on radiologic factors, or linear EBUS imaging findings [35].

### Study limitations

This study is not intended as an examination of diagnostic accuracy of EBUS-TBNA. Given the previously reported specificity of EBUS-TBNA for detection of NSCLC of 100% [36], findings of PET-occult disease will not be performed surgically. Outcomes following systematic LN staging will be reported as a proportion of patients in whom PET-occult metastases are identified by systematic EBUS-TBNA. As surgical confirmation will not be performed, we will not be able to report a true prevalence of PET-occult LN metastases in this group, nor the sensitivity of EBUS-TBNA for detection of PET-occult LN metastases.

While EUS-B-FNA may be performed by pulmonologists, this is dependent on local expertise and institutional credentialing practice. Thus we expect only a minority of patients to have undergone “complete” endoscopic mediastinal staging. Furthermore, PET staging will include assessment of all LN stations, while endoscopic staging will be limited only to LN stations accessible by the technique. EBUS is unable to assess Stations 5, 6, 8 and 9.

Prediction models estimating the probability of nodal disease have been validated [28] and may be valuable in identifying a subset of patients with higher probability of downstaging to early stage (resectable) NSCLC. However, in these models patients with N2 and N3 are grouped together. This study will examine outcomes on a per-lymph node basis and therefore discrimination between N2 and N3 status is not expected from this model it is unlikely.

While in silico modeling will examine the dosimetric consequences of PET-EBUS discrepancies, this study will not directly compare survival outcomes. Delivery of radiotherapy will be determined by local tumour control board assessments, incorporating both PET and EBUS-TBNA findings.

### Conclusion

While the importance of accurate LN staging, both for prognostication and treatment decision-making, for patients with early stage NSCLC is emphasized in the literature as well as international society guidelines, the same importance is not attached to LN staging in patients with stage III disease undergoing curative-intent therapy, despite the evidence supporting the critical impact this may have on treatment planning and outcomes. We intend that this study provide evidence to support increasing use of this simple and established technique of EBUS-TBNA in this patient group.

## Data Availability

This is a study protocol article and no datasets were generated or analysed for this protocol manuscript. To obtain access to the raw data, you may contact A/Prof Daniel Steinfort at Daniel.steinfort@mh.org.au.

## Abbreviations

EBUS: Endobronchial ultrasound
EUS: Endoscopic ultrasound
EUS-B: Endoscopic ultrasound staging
EUS-B-FNA: Endoscopic ultrasound with bronchoscope-guided fine needle aspiration
TBNA: Transbronchial needle aspiration
FNA: Fine needle aspiration
LN: Lymph node
PET: Positron emission tomography
FDG-PET: Fluorodeoxyglucose positron emission tomography
NSCLC: Non-small cell lung cancer
ROSE: Rapid on-site cytology examination
